# Molecular characterization of an adiponectin receptor homolog in the white leg shrimp, *Litopenaeus vannamei*

**DOI:** 10.7717/peerj.2221

**Published:** 2016-07-14

**Authors:** Ah Ran Kim, Md Jobaidul Alam, Tae-ho Yoon, Soo Rin Lee, Hyun Park, Doo-Nam Kim, Doo-Hae An, Jae-Bong Lee, Chung Il Lee, Hyun-Woo Kim

**Affiliations:** 1 Interdiciplinary Program of Biomedical Engineering, Pukyong National University, Busan, South Korea; 2 Department of Marine Biology, Pukyong National University, Busan, South Korea; 3 Department of Marine Bioscience, Gangneung-Wonju National University, Gangneung, Republic of Korea; 4 Korea Polar Research Institute, Korea Ocean Research and Development Institute, Incheon, Republic of Korea; 5 Distant-Water Fisheries Resources Research Division, National Institute of Fisheries Science, Busan, Republic of Korea

**Keywords:** GPCR, RNAi, Adiponectin receptor, Decapod crustacean, Metabolism

## Abstract

Adiponectin (AdipoQ) and its receptors (AdipoRs) are strongly related to growth and development of skeletal muscle, as well as glucose and lipid metabolism in vertebrates. Herein we report the identification of the first full-length cDNA encoding an AdipoR homolog (Liv-AdipoR) from the decapod crustacean Litopenaeus vannamei using a combination of next generation sequencing (NGS) technology and bioinformatics analysis. The full-length Liv-AdipoR (1,245 bp) encoded a protein that exhibited the canonical seven transmembrane domains (7TMs) and the inversed topology that characterize members of the progestin and adipoQ receptor (PAQR) family. Based on the obtained sequence information, only a single orthologous AdipoR gene appears to exist in arthropods, whereas two paralogs, AdipoR1 and AdipoR2, have evolved in vertebrates. Transcriptional analysis suggested that the single Liv-AdipoR gene appears to serve the functions of two mammalian AdipoRs. At 72 h after injection of 50 pmol Liv-AdipoR dsRNA (340 bp) into *L. vannamei* thoracic muscle and deep abdominal muscle, transcription levels of Liv-AdipoR decreased by 93% and 97%, respectively. This confirmed optimal conditions for RNAi of Liv-AdipoR. Knockdown of Liv-AdipoR resulted in significant changes in the plasma levels of ammonia, 3-methylhistine, and ornithine, but not plasma glucose, suggesting that that Liv-AdipoR is important for maintaining muscle fibers. The chronic effect of Liv-AdipoR dsRNA injection was increased mortality. Transcriptomic analysis showed that 804 contigs were upregulated and 212 contigs were downregulated by the knockdown of Liv-AdipoR in deep abdominal muscle. The significantly upregulated genes were categorized as four main functional groups: RNA-editing and transcriptional regulators, molecular chaperones, metabolic regulators, and channel proteins.

## Introduction

Adiponectin, also known as AdipoQ, is a polypeptide hormone secreted exclusively by adipose tissue into the blood of vertebrates (Scherer et al., 1995). AdipoQ induces important effects that include stimulating glucose utilization, oxidating fatty acids and improving insulin sensitivity in vertebrates (Kadowaki et al., 2006). Adiponectin receptors (AdipoRs) convey the AdipoQ signals by phosphorylating and activating 5′ AMP-activated protein kinase (AMPK) and downstream acetyl-CoA carboxylase (ACC) in the target cell (Yamauchi et al., 2002). Although AdipoRs exhibit seven conserved transmembrane domains (TMs), AdipoRs are considered members of the progestin and AdipoQ receptors (PAQR) family because of their unique inversed topology, which is distinct from the typical GPCRs (Yamauchi et al., 2003; Zhu et al., 2008). Vertebrates have two paralogs of these receptors, AdipoR1 and AdipoR2. AdipoR1 is mainly expressed in skeletal muscle, whereas AdipoR2 is predominantly identified in liver, and this suggests that each paralog plays a different role in each tissue (Yamauchi et al., 2003).

Although the AdipoR signaling pathway has drawn attention for its medical and industrial importance in vertebrates (Tsuchida et al., 2004), studies on its homologs have been limited. These include investigations in *Bombyx mori* (Zhu et al., 2008), *Drosophila melanogaster* (Kwak et al., 2013) and *Caenorhabditis elegans* (Svensson et al., 2011). Recently, it has been shown that the AdipoR signaling pathway is closely linked to skeletal muscle growth in vertebrates (Qiao et al., 2012; Suzuki, Zhao & Yang, 2008), which suggests that the AdipoR signaling pathway may be involved in regulating muscle growth in shrimp. Currently, no information about the AdipoR gene or its function has been reported in decapod crustaceans.

White leg shrimp, *Litopenaeus vannamei*, is one of the most widely cultured species in shrimp aquaculture industry because of its tolerance to a wide range of salinity and various pathogens (FAO, 2014). Aside from its economic importance, this species has been used to understand various physiological responses in decapod crustaceans (De Oliveira Cesar et al., 2006; Galindo et al., 2009; Li et al., 2007; Pascual et al., 2006; Wang et al., 2009). Until recently, it has been difficult to understand the physiological responses in non-model organisms as comprehensively as those in model systems, mainly due to a lack of DNA sequence information and a lack of molecular tools to change the target gene expression that produces the physiological changes. The recent advancements of next generation sequencing (NGS) technology and RNA interference (RNAi) technique have enabled researchers who study the non-model organisms, including decapod crustaceans, to expand their knowledge by using those techniques with a limited budget. For example, transcriptional suppression of target gene can be achieved simply by injecting long dsRNA, and this method has changed the strategy for studying crustacean physiology, including growth and development (De Santis et al., 2011; Glazer et al., 2010; Lee et al., 2015; Soñanez Organis, Racotta & Yepiz-Plascencia, 2010), immunity (Robalino et al., 2007), and reproduction (Nagaraju, Rajitha & Borst, 2011; Sathapondecha et al., 2011; Treerattrakool, Panyim & Udomkit, 2011).

In this study, we identified the full-length cDNA that encodes a homolog of mammalian AdipoR (Liv-AdipoR) from the white leg shrimp, *L. vannamei*, by screening an RNA-seq database and performing bioinformatics analysis. The primary structure and transcriptional characters of the receptor were then analyzed. In order to estimate its biological function in skeletal muscle, RNA interference (RNAi) technique was applied in deep abdominal muscle and its effects were analyzed based on glucose and amino acid levels. Transcriptomic change induced by dsRNA injection was also analyzed to determine what kinds of biological pathways might be involved in the Liv-AdipoR gene.

## Materials and Methods

### Experimental animals

Immature *L. vannamei* of similar size (27.26 ± 4.17 mm carapace length) and body mass (11.87 ± 5.07 g) were purchased from a local seafood market. Prior to the experiments, the shrimp were acclimatized in a circulating aerated seawater tank (10 L) for at least 14 days at 27 °C. An Octopus Diablo DC 170 skimmer (Reef Octopus, Guangdong, China) was used to eliminate nitrogenous waste. The photoperiod was maintained at 12L:12D and shrimp were fed diced squid and polychaetes (5% of body weight). Salinity (34 ± 2 psu) was maintained by addition of deionized water each day and by replacing 20% of the total volume with fresh seawater each week. Molt periods were recorded and molt stage was determined based on the degree of setae development, as described previously (Chan, Rankin & Keeley, 1988).

### Sequence analysis of full-length Liv-AdipoR

Partial *L. vannamei* cDNA sequences exhibiting high similarity to mammalian AdipoRs (JP424300) were originally identified by performing a nucleotide similarity search in the GenBank database (http://www.ebi.ac.uk/Tools/sss/ncbiblast/nucleotide.html). To obtain the remaining coding region and each 5′ and 3′ untranslational region (UTR), rapid amplification of cDNA ends (RACE) was carried out as described previously (Lee et al., 2011). The open reading frame (ORF) and the deduced amino acid sequences were predicted using an ORF finder program (http://www.ncbi.nlm.nih.gov/gorf/gorf.html) and its full length was confirmed by RT-PCR using sequence-specific primers (Table 1) designed with IDTSciTools (http://eu.idtdna.com/analyzer/applications/OligoAnalyzer/). Multiple alignments analysis was performed using the ClustalW2 program (http://www.ebi.ac.uk/Tools/clustalw2/) and these results were represented using GenDoc 2.7 (http://www.nrbsc.org/gfx/genedoc/index.html). The topology of Liv-AdipoR was predicted using the TopPred 1.10 program (Claros & Von Heijne, 1994) and phylogenetic analysis was performed using the MEGA 6 (Molecular Evolutionary Genetics Analysis version 6.0.) program with the Neighbor-Joining method and the 1,000-replicate bootstrap test (Saitou & Nei, 1987; Tamura et al., 2013).

**Table 1:** Primers used for Liv-AdipoR.

Primer name	Sequence (5′–3′)	Description
Liv-AdipoR-F1	TGTTTGATCGACACCATGAG	Forward primer for full-ORF confirm
Liv-AdipoR-R1	CTAAAGGATGTCCTGCGCTTCGAT	Reverse primer for full-ORF confirm
Liv-AdipoR-5RACE-R1	GTGACACGAGACTGTATGGAAG	First reverse primer for 5′ RACE
Liv-AdipoR-5RACE-R2	CAGAAGATGGCACCGATG	Second reverse primer for 5′ RACE
Liv-AdipoR-3RACE-F1	CCTTAGGCTGGCTTATTCTTATG	First forward primer for 3′ RACE
Liv-AdipoR-3RACE-F2	CTTATGGGAGCATTGTATGTCTTG	Second forward primer for 3′ RACE
Liv-AdipoR-RT-F1	TTCGAGACTGCGGAGGAGTTAG	Forward primer for RT-PCR & Real-time
Liv-AdipoR-RT-R1	GGTTGACATCAAGGAGAAGCTC	Reverse primer for RT-PCR & Real-time PCR
Liv-AdipoR-dsRNA-F1	GAATTTAATACGACTCACTATAGGGCCAC CGTTCTATGGCCCAGAGTGCCTTC	Forward primer for dsRNA synthesis
Liv-AdipoR-dsRNA-R1	GAATTTAATACGACTCACTATAGGGCCAC CGCTGTGAATACGAGCTTCTCC	Reverse primer for dsRNA synthesis
18s rRNA F1	CTGCGACGCTAGAGGTGAAATTC	Forward primer for RT-PCR & Real-time PCR
18s rRNA R1	GGTTGCAAAGCTGAAACTTAAAGG	Reverse primer for RT-PCR & Real-time PCR

### Transcriptional analysis of Liv-AdipoR

After the each experiment, shrimp were sacrificed and dissected. The isolated tissues were directly frozen in liquid nitrogen and stored at −80 °C prior to total RNA extraction. Total RNA was isolated from dissected tissues using Trizol Reagent (TaKaRa, Shiga, Japan) according to the manufacturer’s protocol. RNA purity was verified by measuring the absorbance at 260 and 280 nm in an ND-1000 spectrophotometer (NanoDrop Technologies, Wilmington, DE, USA), and RNA integrity was detected by electrophoresis in a 1.0% agarose gel. A clean single band from the “hidden break” of 28s rRNA indicates the integrity of the isolated RNA in decapod crustaceans (Macharia, Ombura & Aroko, 2015). Before reverse transcription, total RNA was treated with DNase I (TaKaRa, Japan) to remove the genomic DNA. The cDNA for each sample was synthesized from an equal amount of total RNA (1,000 ng) by M-MLV reverse transcriptase (Invitrogen Co., Carlsbad, CA, USA) following the manufacturer’s protocol with random hexamer primer.

A tissue distribution profile was obtained by end-point RT-PCR using cDNAs from various tissues, including the gills, hemocyte, epidermis, hepatopancreas, gonad, brain, thoracic ganglia, heart and muscle. Sequence-specific primers for 360 bp of Liv-AdipoR transcript and for 254 bp of 18s rRNA were used (Table 1). After qPCR, melting temperature (Tm) analysis showed single peak and a single PCR band was identified, indicating that both primers were suitable for further experiments. The RT-PCR programming was 94 °C for 5 min followed by 35 cycles at 94 °C for 30 s, 60 °C for 30 s, 72 °C for 30 s, and a final cycle of 72 °C for 5 min. The PCR products were separated on a 1.5% agarose gel stained with ethidium bromide.

The transcriptional level of Liv-AdipoR was measured by qRT-PCR in a DNA Engine Chromo4 real-time Detector (Bio-Rad, Hercules, CA, USA). Efficiencies of the PCR reactions were calculated as described previously (Kim et al., 2012). The PCR reactions were carried out in 20 µL reaction systems with 10 µL 2×SYBR^®^ Premix Ex Taq™ II (TaKaRa, Japan), 1 µL forward primer (10 pmol), 1 µL reverse primer (10 pmol), 500 ng of cDNA template, and 3 µL sterile distilled water. Thermal cycling conditions were 95 °C for 30 s, followed by 40 cycles of 95 °C for 5 s, 60 °C for 30 s, and 72 °C for 30 s. Data were statistically analyzed by one-way analysis of variance (one-way ANOVA) using the Minitab 16 Statistical software (Minitab Inc., State College, PA, USA). Group results were compared using Student’s *t*-test (Microsoft Excel ver. 2013). Differences were considered to be significant at *p* < 0.05.

### RNA interference of Liv-AdipoR

The target sequence for Liv-AdipoR interference was determined using SciTools RNAi design software (http://sg.idtdna.com/Scitools/Applications/RNAi/RNAi.aspx). Different from the system for the mammalian RNAi, use of long segments of double-stranded RNA (dsRNA) has been successful for knocking down target genes in decapod crustacean systems (Lee et al., 2015; Lugo et al., 2006; Sagi, Manor & Ventura, 2013). Using this method, 340 bp of Liv-AdipR dsRNA was synthesized according to the procedure described previously (Lee et al., 2015). Briefly, after a template was generated by sequence-specific forward and reverse primers designed to include a T7 promoter extension at the 5′ end (Table 1), cRNA was transcribed using the mMESSAGE mMACHINE Kit (Ambion Inc., USA) and purified using the RNeasy Mini Kit (Qiagen Inc., Hilden, Germany). To achieve precise annealing of the synthesized dsRNA, RNA samples were subjected to the following conditions: denaturation at 95 °C for 5 min and annealing by gradually lowering the temperature (1 °C every 30 s) from 95 °C to 25 °C. The integrity and quantity of newly synthesized dsRNA were determined using 1% agarose/ethidium bromide gel electrophoresis and using an ND-1000 spectrophotometer. RNA stock solutions were aliquoted and stored at −80 °C prior to use.

The dsRNA was injected by syringe (with 0.3-mm G, 8-mm needle) into the deep abdominal muscle between the second and third pleopods of each shrimp. For the short-term experiment, seven shrimp received 10 pmol dsRNA and eight shrimp received 50 pmol dsRNA Three days later, all of these shrimp were dissected and stored at −80 °C. For the long-term experiment, the body weight and carapace length of twenty four shrimp were measured initially, and then 50 pmol dsRNA was injected weekly into fourteen shrimp (experimental group) and 30 µL of 1X phosphate-buffered saline without dsRNA was injected into ten control shrimp. The mortality and molting frequency were recorded for each group daily.

### Transcriptomic analysis of Liv-AdipoR knockdown

To estimate the physiological effects of skeletal muscle induced by Liv-AdipoR knockdown, transcriptomes of shrimp injected with dsRNA injection were compared with those of the control group using RNA-seq strategy. At 3 days post-injection, total RNA was extracted from the deep abdominal muscles of eight individuals in each group and the RNA was pooled. The quantity and quality of total RNA were measured using a Qubit Fluorometer (Life Technologies, Carlsbad, CA, USA) and Agilent 2100 Bioanalyzer (Agilent Technologies, Santa Clara, CA, USA). cDNA libraries were prepared with 2 µg of total RNA using the TruSeq^®^ Sample Preparation V2 (Illumina, San Diego, CA, USA) according to the manufacturer’s instructions. Constructed cDNA libraries were then sequenced on the Miseq System platform (Illumina) using 150*2 paired-end reads.

The reference transcriptome database was constructed using reads obtained in this study and five RNA-seq data (eyes, stomach, heart, hepatopancreas, whole head) from Genbank under Biosample SAMN02863073. Raw sequencing outputs were imported into the CLC Genomics Workbench 7.5 environment (CLC Bio Aarhus, Denmark) and trimmed using a base caller quality threshold of 0.05 and 200 nucleotides. The *de novo* transcriptome assembly was performed with default setting to produce contigs with 200 nucleotides. Each contig was functionally annotated by BLASTX search against the non-redundant (nr) protein database with an e-value threshold to 10^–3^. Associated Gene Ontology (GO) and Kyoto Encyclopedia of Genes and Genomes (KEGG) annotation were assigned using the Blast2Go program with results of the BLASTX search.

The relative transcriptional levels were analyzed using CLC Genomics workbench software 8.0. The reads from the experimental and control groups were mapped against the reference transcriptome database generated by *de novo* assembly. The expression level of each contig was represented as reads per kilobase per million mapped reads (RPKM). Baggerley’s tests were performed to identify differentially expressed genes by comparing normalized gene reads between the two groups. We considered a Baggerley’s *P*-value less than 0.05, an RPKM ratio more than twofold, and surpassing ±10 normalized fold change values between the two groups to indicate differentially expressed genes (DEGs).

### Glucose and amino acid assay in hemolymph

Changes in hemolymph glucose and free amino acids were measured at 3 days post-injection. Hemolymph was collected from the ventral sinus at the base of the first abdominal segment using a 1-mL syringe. The hemolymph was mixed with half its volume of EDTA (0.05 M) and then centrifuged at 3,000×g for 10 min. The supernatant was recovered. Glucose levels in the supernatant were measured using a FUJIFILM DRI-CHEM NX500i machine (FujiFilm, Tokyo, Japan) and FUJI DRY CHEM SLIDES GLU-P III kit (FujiFilm, Tokyo, Japan), and free amino acids were measured in a Hitachi High-Speed Amino Acid Analyzer L-8900 (Hitachi High-Technologies, Tokyo, Japan). All measurements were recorded in triplicate. Data were statistically analyzed using Student’s *t*-test (Microsoft Excel ver. 2013). Differences were considered to be significant at *p* < 0.05.

**Figure 1:**
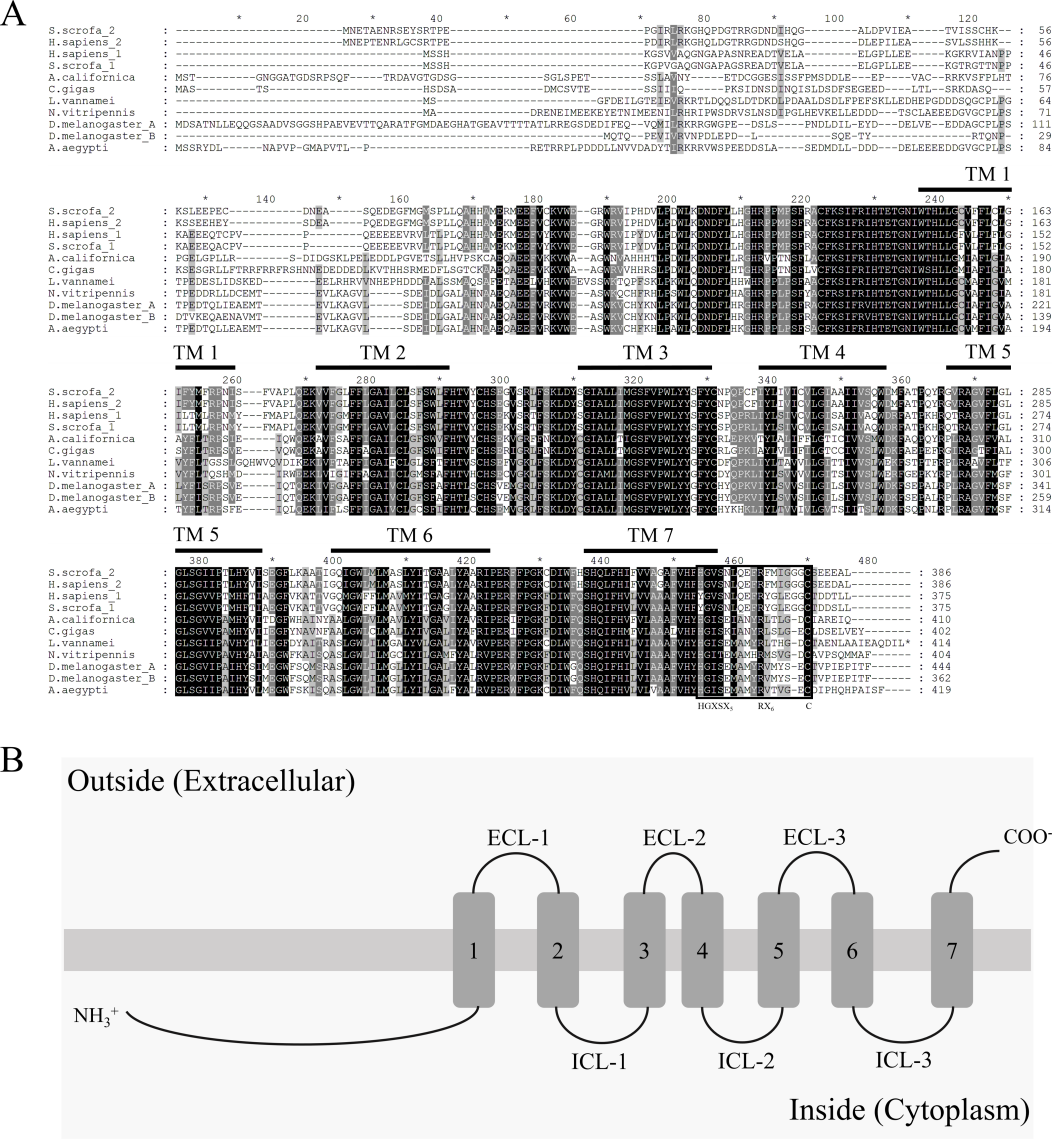
Multiple alignments analysis and topology prediction. (A) Multiple alignment of AdipoRs in various taxa. Conserved amino acid residues were shaded in black (100%) and in grey (above 60%). Seven transmembrane regions shown by the overbars. The conserved residues (HGXSX_5_RX_6_C) within the C-terminal region were boxed. The GenBank accession number : *Homo sapiens 1*, NP_116054; *Homo sapiens 2*, NP_078827; *Sus scrofa 1*, AAT72305; *Sus scrofa 2*, NP_001007193; *Aplysia californica*, XP_005097206; *Crassostrea gigas*, XP_011439974; *Drosophila melanogasterA*, NP_651061; *Drosophila melanogasterB*, NP_732759; *Aedes aegypti*, EAT33030; *Nasonia vitripennis*, NP_001153422 (B) Predicted topology of Liv-AdipoR. Topology was predicted by two programs TopPred 1.10 (http://bioweb.pasteur.fr/seqanal/interfaces/toppred.html) and HMMTOP 2.0 (http://topcons.cbr.su.se/). Predicted structure was depicted by the Microsoft PowerPoint (Microsoft, ver. 2013). ICL 1-3, Intracellular loops 1-3; ECL 1-3, Extracellular loops 1-3.

**Figure 2:**
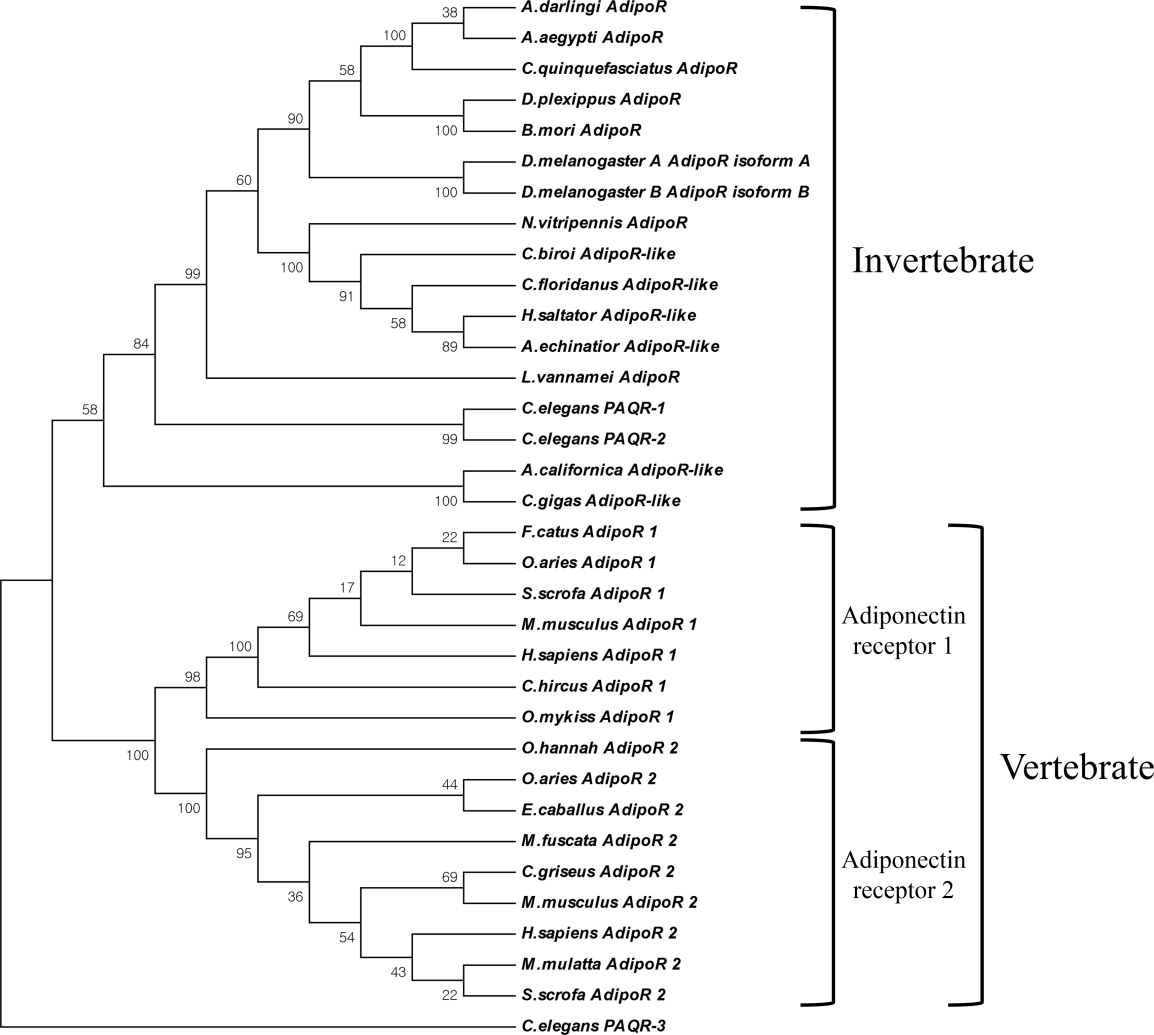
Phylogenetic analysis of AdipoRs. The phylogenetic tree was constructed by the Neighbor-Joining algorithm with 1,000 replicates of bootstrap using MEGA 6 (Molecular Evolutionary Genetics Analysis version 6.0.) program. The GenBank accession number : *Homo sapiens 1*, NP_116054; *Homo sapiens 2*, NP_078827; *Sus scrofa 1*, AAT72305; *Sus scrofa 2*, NP_001007193; *Ovis aries 1*, NP_001293039; *Ovis aries 2*, AHK05782; *Macaca mulatta 2*, NP_001253547; *Macaca fuscata 2*, BAG16754; *Mus musculus 1*, AAH14875; *Mus musculus 2*, NP_932102; *Oncorhynchus mykiss 1*, NP_001268274; *Felis catus 1*, BAG68817; *Ophiophagus hannah 2*, ETE74039; *Capra hircus 1*, NP_001272659; *Cricetulus griseus 2*, ERE67160; *Equus caballus 2*, NP_001157302; *Nasonia vitripennis 1*, NP_001153422; *Drosophila melanogasterA*, NP_651061; *Drosophila melanogasterB*, NP_732759; *Culex quinquefasciatus 2*, XP_001844362; *Aedes aegypti*, EAT33030; *Bombyx mori*, NP_001093316; *Aplysia californica*, XP_005097206; *Crassostrea gigas*, XP_011439974; *Harpegnathos saltator*, EFN77328; *Acromyrmex echinatior*, EGI68824; *Camponotus floridanus*, EFN60769; *Cerapachys biroi*, EZA53550; *Danaus plexippus*, EHJ69172; *Anopheles darling*, ETN58701; *Caenorhabditis elegans-1*, NP_001293733; *Caenorhabditis elegans-2*, NP_498148; *Caenorhabditis elegans-3*, NP_502745.

## Results

### Cloning and structural analysis of Liv-AdipoR

As a result of screening the database and applying a PCR-based cloning strategy, the first crustacean cDNA of the Liv-AdipoR homolog Liv-AdipoR (GenBank number: AKV16260) was identified from *L. vannamei*. Full-length Liv-AdipoR cDNA (1,245 bp) encoded a protein with 414 amino acid residues (Fig. 1A) and showed high similarity to homologs from insect species, including *Zootermopsis nevadensis* (KDR17851, 67%) and *Bombyx mori* (NP_001093316, 67%). Multiple-amino-acid alignment was performed to compare structural similarity with AdipoRs in different species (Fig. 1A). As shown in other homologs in various taxa (Yamauchi et al., 2014), Liv-AdipoR is composed of a long and variable N-terminal region, the highly conserved seven TM, and a relatively conserved short C-terminal region with four residues that are responsible for binding a zinc ion and HGXSX_5_RX_6_C motif (Zhu et al., 2008). Topology prediction analysis showed that the longer N-terminal region and three intracellular loops (ICL-1-3) face the cytoplasm, whereas three extracellular loops (ECL1-3) and the short C-terminal region face the cell exterior outward (Fig. 1B). This is unique to PAQR family members and is distinct from the canonical GPCRs, which supports the idea that Liv-AdipoR belongs to crustacean PAQR members. Phylogenetic analysis showed two major clades, AdipoRs in invertebrates (including Liv-AdipoR), and AdipoRs in vertebrates (Fig. 2). In vertebrates, AdipoR1 and AdipoR2 were clustered in each group, suggesting that the gene duplication event occurred before their evolution within the vertebrate lineage. Instead, only a single copy of AdipoR gene has been identified in invertebrates including mollusks and arthropods. Although three AdipoR genes were identified in the nematode *Caenorhabditis elegans*, AdipoR3 was not orthologous to those in other invertebrates, and both AdipoR1 and AdipoR2 were revealed to be the ancestral genes of other invertebrate AdipoRs, suggesting that a gene duplication event occurred only within the nematode species independent from other invertebrate taxa (Fig. 2). Additionally, two copies of AdipoRs were also identified in *Drosophila melanogaster*, but these were isoforms produced by alternative transcription from a single gene (Fig. 2). Collectively, arthropods appears to possess only a single AdipoR gene.

### Expression analysis of Liv-AdipoR

**Figure 3:**
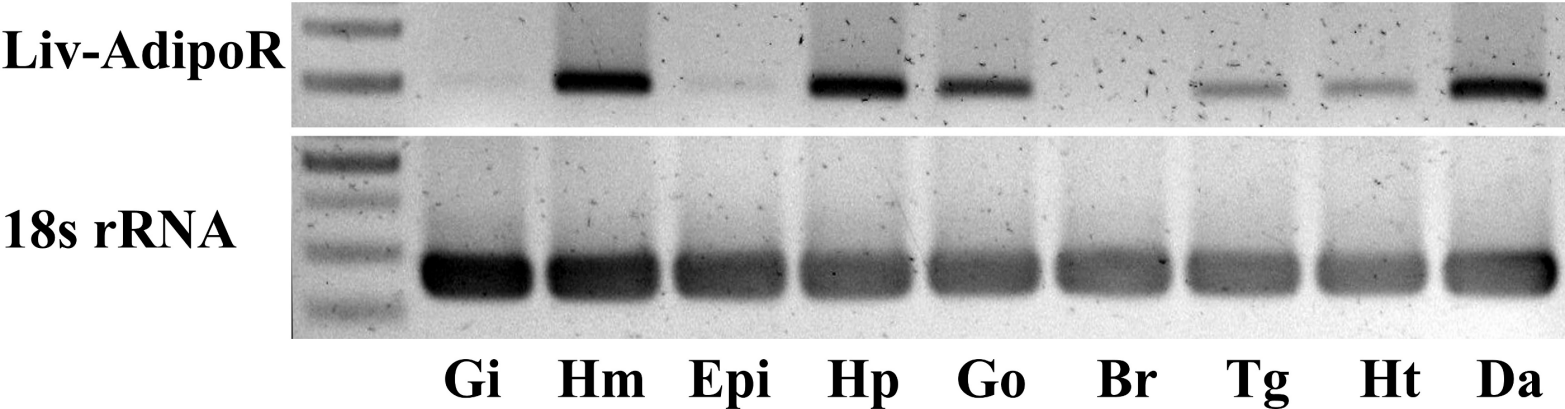
Tissue distribution profile of Liv-AdipoR. Inversed image of PCR products separated on 1.5% agarose gel. The 18S rRNA was used as a control. M, size marker; Gi, gill; Hm, hemocyte; Epi, epidermis; Hp, hepatopancreas; Go, Gonad; Br, brain; Tg, thoracic ganglia; Ht, heart; Da, Deep abdominal muscle.

**Figure 4:**
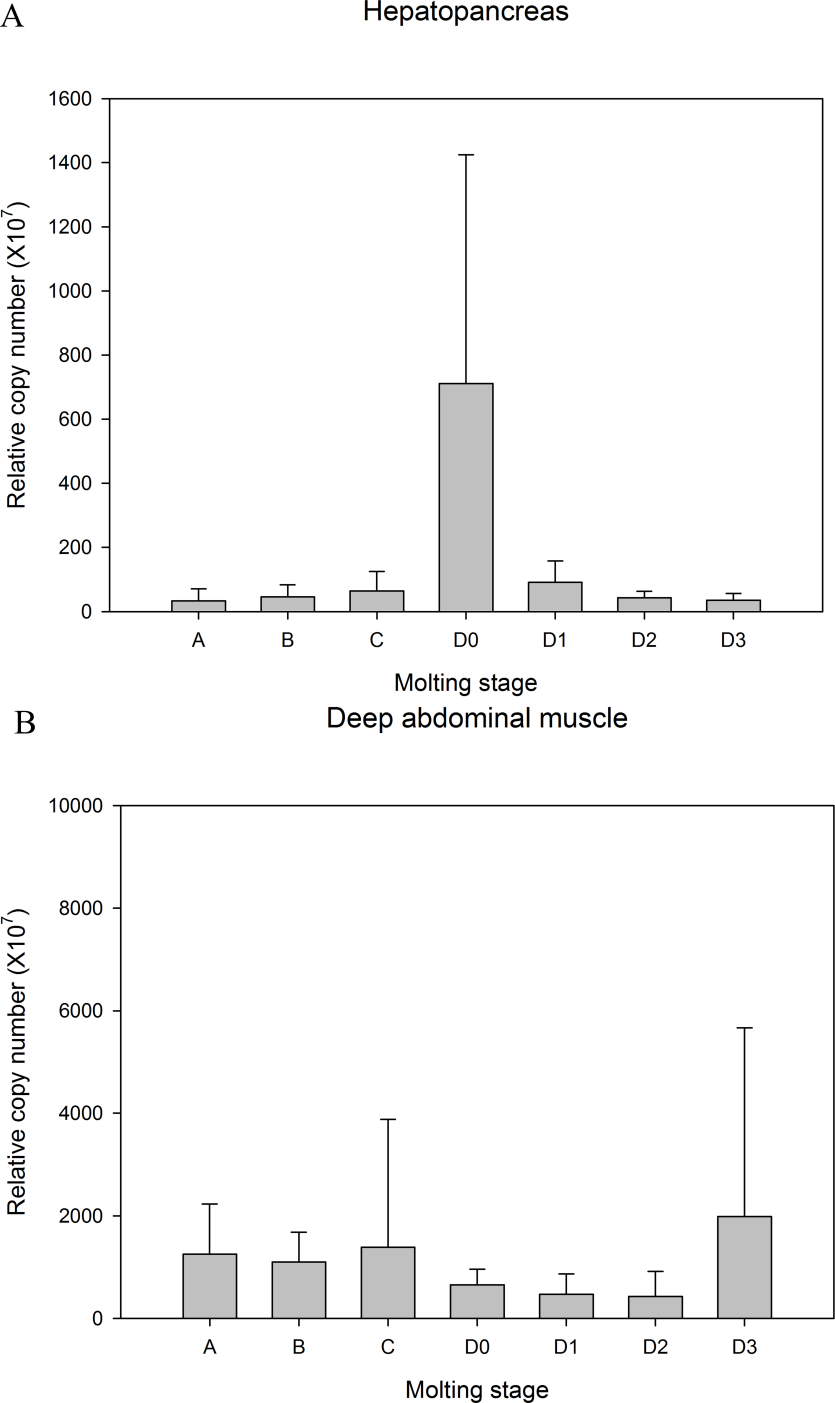
Relative copy number of Liv-AdipoR in (A) hepatopancreas and (B) deep abdominal muscle in different molt cycle. Copy numbers were normalized by the number of 18S rRNA. Stage A (early post molt), Stage B (late postmolt), Stage C (intermolt), Stage D_0_ (onset of premolt), Stage D_1_ (early premolt) Stage D_2_ (intermediate premolt), Stage D_3_ (late premolt).

Major production sites for Liv-AdipoR included hemocytes, the hepatopancreas, gonad and deep abdominal muscle, and its transcripts were also detected in the thoracic ganglia and heart (Fig. 3). Much lower expression was identified in the epidermis and gill, and no detectable transcription of Liv-AdipoR was identified in the brain (Fig. 3). To investigate whether expression of Liv-AdipoR is linked to molt cycle, qPCR was performed in deep abdominal muscle and hepatopancreas tissue from shrimp at different molt stages (Figs. 4A and 4B). No statistical changes were observed across the molt cycle in either of these tissues; however, we did identify individuals with considerably high levels of Liv-AdipoR transcript in the hepatopancreas during D_0_ stage (Fig. 4A).

### RNAi of Liv-AdipoR by injecting dsRNA

**Figure 5:**
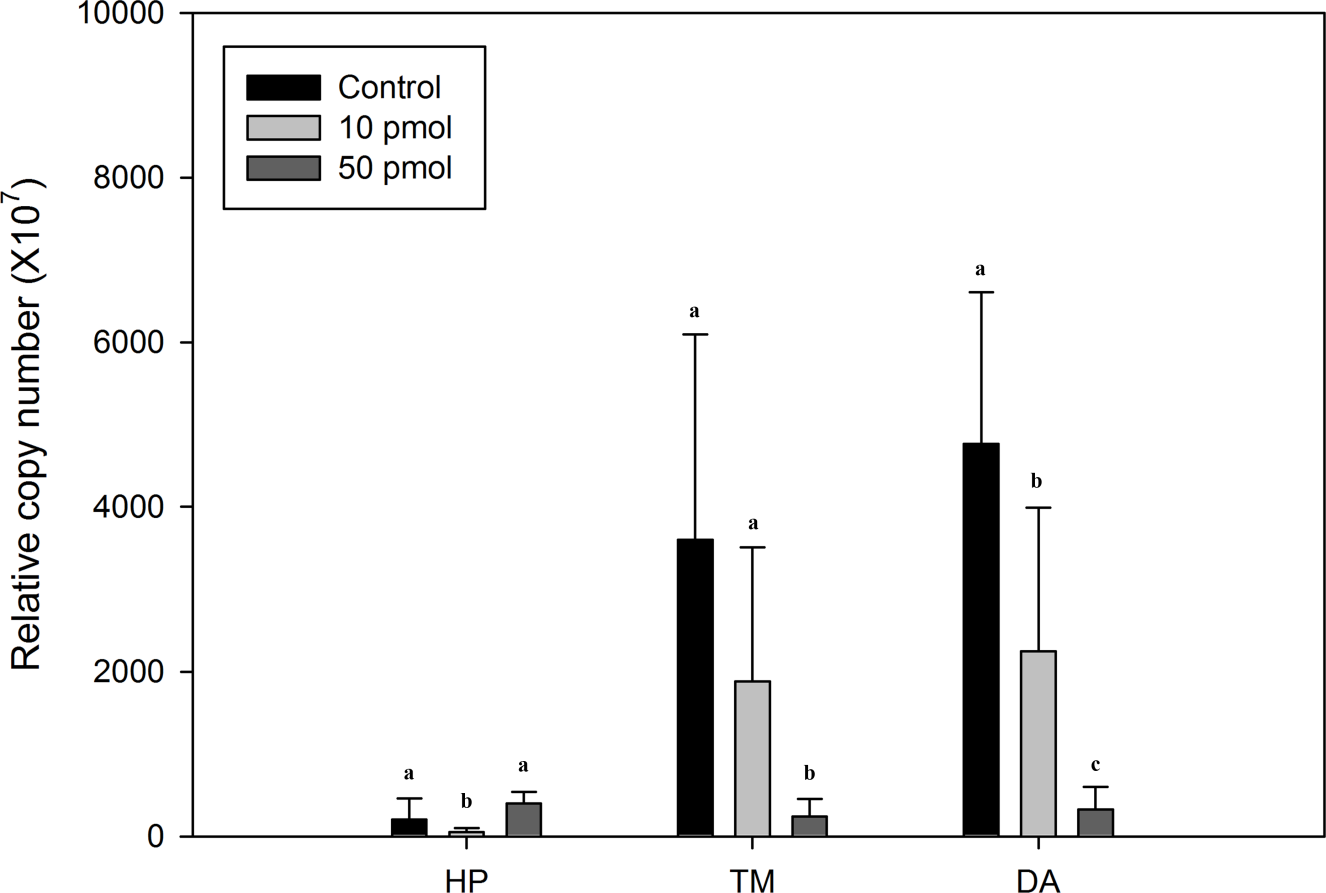
Comparison of Liv-AdipoR transcripts before and after its long dsRNA injection. Transcription level was measured in three tissues including hepatopancreas (HP), thoracic muscle (TM) and deep abdominal muscle (DA) 3 days after dsRNA injections at deep abdominal muscle with three different concentrations (1× PBS, 10 pmol, 50 pmol). Copy numbers were normalized by the number of 18S rRNA. Statistical difference (*P* < 0.05) are shown in different letters.

To estimate the physiological functions of Liv-AdipoR in *L. vannamei*, RNAi technique was applied. This revealed that the effect of dsRNA injection on transcription of Liv-AdipoR differed between the hepatopancreas and skeletal muscle (Fig. 5). Seventy-two hours after injecting long dsRNA into the Liv-AdipoR gene, we failed to observe consistent reduction of the gene’s transcript in the hepatopancreas. Specifically, expression in this tissue decreased approximately 74% after 10 pmol injection, whereas no significant reduction was observed in shrimp that received 50 pmol injection. In contrast, dose-dependent reduction of Liv-AdipoR transcripts was observed in muscle tissue (Fig. 5). Seventy-two hours after 10 pmol dsRNA injection, 80% and 52% reductions of Liv-AdipoR transcripts were identified in thoracic muscle and deep abdominal muscle, respectively. In the shrimp that received 50 pmol dsRNA injection, the corresponding reductions were 97% and 93%.

There was no significant difference in glucose level in hemolymph in the control shrimp (15.818 mg/dL ± 2.724) compared to the dsRNA-injected group (14.286 mg/dL ± 4.165). In contrast, we did observe a difference for free amino acids in hemolymph (Table 2). Compared with findings in the control group, NH_3_ and ornithine were significantly greater in the dsRNA-injected group and 3-methylhistine (3-MeH) was detected only in the dsRNA-injected group. Four weeks after the Liv-AdipoR knockdown experiment, there was a significant difference in mean survival days between the dsRNA-injected group and the control group (15.7 days [14.3% survival rate] versus 25.6 days [40% survival rate], respectively).

**Table 2:** Comparison of free amino acids between control and Liv-AdipoR knockdown shrimp.

Name	Control (Conc/ng)	50 pmol (Conc/ng)
P-Ser	17.019 ± 3.181	25.05 ± 14.3
Tau	385.991 ± 134.83	619.128 ± 83.349
Urea	1353.942 ± 75.824	2019.922 ± 923.513
Asp	4.024 ± 4.759	62.967 ± 51.849
Thr	82.653 ± 13.153	50.276 ± 19.259
Ser	52.956 ± 7.801	149.881 ± 51.532
Glu	714.838 ± 128.779	782.763 ± 334.174
Gly	174.094 ± 55.212	368.48 ± 123.308
Ala	230.566 ± 19.556	322.171 ± 111.371
a-ABA	6.832 ± 0.471	9.447 ± 2.302
Val	77.518 ± 13.203	104.093 ± 35.411
Cys	47.435 ± 5.857	42.623 ± 16.767
Met	39.911 ± 7.618	38.498 ± 12.405
Cysthi	30.949 ± 3.923	31.41 ± 16.329
Ile	47.259 ± 9.645	46.188 ± 13.52
Leu	94.006 ± 25.507	96.613 ± 15.281
Tyr	1.366 ± 1.932	7.515 ± 8.973
Phe	55.14 ± 6.131	82.535 ± 45.067
b-Ala	6.48 ± 4.922	3.843 ± 5.435
NH_3_	37.989 ± 2.726	92.245 ± 25.687^*^
Orn	4.416 ± 1.992	17.857 ± 6.062^*^
Lys	229.253 ± 76.073	144.053 ± 27.886
His	40.519 ± 2.904	62.683 ± 21.077
3-MeH	N/D	8.897 ± 3.515^*^
Arg	276.101 ± 33.477	363.224 ± 111.347
Pro	497.175 ± 145.178	878.617 ± 351.817

**Notes.**

* indicates *p* < 0.05.

N/D for not detected

**Table 3:** Summary of upregulated genes in DA muscle induced by dsRNA injection.

Feature ID	Sequence description	Accession number (coverage)	Fold change	E-value
**RNA-editing and transcriptional regulators**				
2010	DNA mismatch repair protein MutS	WP_041915889 (24%)	224.54	1e–04
12231	RNA-directed DNA polymerase from mobile element jockey	KFM66762 (13%)	175.38	1e–25
662	Argonaute 2	ADK25181 (94%)	64.75	0.0
3212	NFX1-type zinc finger-containing protein 1-like	KPP76956 (30%)	38.25	4e–31
7396	RNA exonuclease 4	EKC41786 (59%)	21.51	1e–61
7988	DNA helicase	KZS08746 (90%)	20.88	0.0
17085	Pre-mRNA-splicing factor ATP-dependent RNA helicase PRP16	KFM78446 (59%)	17.74	0.003
18842	Enhancer of yellow 2 transcription factor-like protein	EOB01110 (33%)	17.10	7e–33
**Molecular chaperones**				
2976	E3 ubiquitin-protein ligase TTC3	KXJ25685 (18%)	32.82	5e–13
562	probable E3 ubiquitin-protein ligase DTX2	XP_008493267 (47%)	25.68	7e–66
11991	E3 ubiquitin-protein ligase TRIM39-like	XP_007070626.1 (20%)	14.96	3e–09
21720	polyubiquitin isoform X2	XP_010121053 (77%)	12.67	9e–20
5125	E3 ubiquitin-protein ligase TRIM32	NP_001248279 (20%)	12.05	7e–12
8317	Heat shock protein 90	AGC54636 (74%)	10.46	0.0
**Metabolic regulators**				
6271	Granulins-like isoform X3	XP_009862364 (54%)	22.60	1e–96
29409	putative Proline dehydrogenase	KZS13984 (39%)	19.55	2e–91
2112	Carboxyl/choline esterase	AIY69041 (100%)	18.85	1e–11
17840	5′-nucleotidase	KDR18455 (53%)	18.11	0.0
8958	Triosephosphate isomerase	ADG86240 (55%)	17.96	6e–100
23308	Hemocyte homeostasis-associated protein	ADN43412 (36%)	17.53	2e–16
18318	Dihydropteridine reductase	KDR08654 (24%)	17.34	1e–89
1435	Alanine racemase	BAH22617 (31%)	15.82	0.0
10775	Peptidyl-prolyl cis-trans isomerase	KOB74010 (34%)	14.96	3e–30
43837	UMP-CMP kinase-like Protein	EFA11131 (66%)	11.88	2e–60
21361	poly [ADP-ribose] polymerase	NP_766481 (81%)	11.32	6e–54
**Channels and receptor proteins**				
9949	Sugar transporter	ETN65997 (40%)	81.68	3e–34
52876	Mitochondrial ornithine transporter 1	KZS04188 (39%)	80.94	6e–122
20165	Equilibrative nucleoside transporter 3	KDR21110 (32%)	42.62	1e–84
12695	Transient receptor potential cation channel subfamily A member 1	KXJ09705 (76%)	37.70	1e–55
**Unclassified**				
2550	prohormone-1	ALQ28598 (37%)	39.36	1e–23

**Table 4:** Summary of downregulated gene in DA muscle induced by dsRNA injection.

Feature ID	Sequence description	Accession number (coverage)	Fold change	E-value
**Immune response**				
39468	Masquerade-like protein	CAA72032 (77%)	–194.79	3e–134
16844	Cytochrome P450 CYP379A1	ACI94903 (97%)	–27.95	1e–19
19867	Cuticle protein 6	P82119 (73%)	–24.34	8e–24
4780	Heat shock protein 21	AET34915 (68%)	–27.62	8e–13
1917	Heat shock protein 21	AET34915 (97%)	–23.44	6e–11
2619	Heat shock protein 21	AET34915 (67%)	−21	2e–23
2686	Heat shock protein 21	AET34915 (43%)	–16.59	5e–40
46865	Ribosomal protein L7	AFU93449 (75%)	–16.57	8e–93
**Transcription factor**				
8234	Kruppel-like protein 1	AEW22981 (16%)	–142.78	1e–35
18410	Protein msta, isoform A	KDR21630 (72%)	–52.3	1e–04
**Others**				
29645	Beta-N-acetylglucosaminidase	ACR23316 (56%)	–86.57	4e–86
17352	Beta-N-acetylglucosaminidase	AFZ76982 (77%)	–34.26	2e–38
33455	Collagen alpha chain, type IV	XP_002409121 (87%)	–18.93	7e–30
19255	Liv-AdipoR	AKV16260	–11.22	0

### Transcriptomic analysis after Liv-AdipoR dsRNA injection

To estimate the physiological responses induced by the Liv-AdipoR knockdown, we compared transcriptomes before and after dsRNA injection (Tables 3 and 4). Since suppression of the Liv-AdipoR transcript was most effective in the deep abdominal muscle injected with 50 pmol of dsRNA, transcriptomes of deep abdominal muscles with and without dsRNA injection were compared. Using the Illumina MiSeq platform, 61.26 million reads of average length 75.3 bp were generated from the shrimp cDNA library. After trimming and filtering, 61.24 million quality-trimmed sequences from five SRA files were de novo assembled. The 53,029 contigs generated by the assembly process ranged from 200 to 24,286 bp, with 2,094 bp of N50 value (average contig length, 1,138 bp). Of all the contigs generated, 18,686 transcripts provided at least one BLASTX hit against the Nr data with an e-value < 10–5. Of the 53,029 contigs, 1,016 were transcriptionally changed (804 upregulated and 212 downregulated). Of the 804 upregulated contigs, 42 (Table 3) were ultimately determined to be reliable genes after contigs with low sequence similarity (e-value < 10–5), high *P*-value (>0.05), and lower degrees of change (<10 fold change) were eliminated.

The decreased number of Liv-AdipoR transcripts (i.e., 11.23-fold reduction) was reconfirmed, and this was similar to the qPCR result (95%), which indicated that the knockdown was successful (Table 4). The upregulated genes were able to be classified in four major categories of cellular functions: RNA-editing and transcriptional regulators, molecular chaperones, metabolic regulators, and channel and receptor proteins. ID2010, ID12231, ID662, ID3212, ID7396, ID7988, ID17085, and ID18842 belonged to RNA-editing and transcriptional regulators. These were among the highest upregulated genes, and the identification of RNA-directed DNA polymerase from transposon X-element and argonaute 2 (Ago2) supported that the injected dsRNA induced RNAi, and that those genes could be further used not only as positive controls for later RNAi experiments, but also for understanding the mechanism of RNAi in decapod crustaceans. Molecular chaperones, including ID2976, ID562, ID11991, ID5125, ID8317, and ID21720, were among the next highest upregulated genes (Table 3). The metabolic regulators included ID6271, ID29409, ID2112, ID17840, ID8958, ID23308, ID18318, ID1435, ID10775, ID43837, and ID21361. The finding of granulin-like proteins in the decapod crustacean was of interest, as these are known to regulate cell growth. Finally, various channel and receptor proteins were upregulated, including a sugar transporter (ID9949), mitochondrial ornithine transporter1 (ID52876), nucleotide transporter 3 (ID20165), and cation channel subfamily A member 1 (ID12695), which suggested that the knockdown Liv-AdipoR was involved with transportation of certain types of carbohydrates, amino acids, and nucleotides, as well as ion exchange. It is also noteworthy that injection of Liv-AdipoR dsRNA into skeletal muscle caused significant induction of one prohormone-1 (ID2550), and this should be investigated further. Compared with upregulated genes, a relatively smaller number of contigs were identified as downregulated genes (Table 4). Eight of these contigs were involved with immune response, including a masquerade-like protein, heat shock protein 21, and ribosomal protein L7. Two transcription factors and two chitin-degrading proteins were also downregulated, and this should be analyzed further.

## Discussion

In this study, we isolated and characterized the full-length cDNA that encodes Liv-AdipoR in the shrimp *L. vannamei*, and our findings indicate that the AdipoR signaling pathway exists in decapod crustaceans. In vertebrates, AdipoQ and AdipoR signaling induces skeletal muscle biogenesis not only through increased fatty acid uptake and oxidation and suppressed fatty acid synthesis, but also through improved mitochondrial bioenergetics (Qiao et al., 2012; Ritchie & Dyck, 2012; Yoon et al., 2006). It is reasonable to suggest that understanding Liv-AdipoR and its signaling pathway can be useful for identifying regulators of crustacean muscle growth. In vertebrates, functional suppression of myostatin (MSTN), a negative regulator of skeletal muscle growth, was found to upregulate the AdipoR signaling pathway and also enhance muscle growth (Suzuki, Zhao & Yang, 2008). Recently, a MSTN homolog was also isolated and characterized in decapod crustaceans (Covi, Kim & Mykles, 2008; Lee et al., 2015; MacLea et al., 2010). Understanding the relationship between AdipoR and the MSTN signaling pathways would help expand our knowledge of muscle growth and development in decapod crustaceans.

The isolated Liv-AdipoR featured the canonical characters of PAQR family members, including conserved seven TM, a long internal N-terminal region, and a relatively short external C-terminal region. Based on the arthropod AdipoRs that are currently known, including the Liv-AdipoR we identified in this study and two from insect species *B. mori* (Zhu et al., 2008) and *D. melanogaster* (Kwak et al., 2013), deduced amino acids and their structures are highly conserved, and this suggests that their ligands and signaling pathways may also be conserved in arthropods. However, the homolog of adiponectin has not yet been identified in arthropods despite numerous insect genome data that suggest ligands for AdipoRs in arthropods may be different from those for AdipoQ in vertebrates. Although the HGXSX5RX6C motif at the C-terminal region, which has been known as the ligand binding site, is well conserved in all AdipoRs that have been compared (Zhu et al., 2008), recently published crystal structures of human AdipoR1 and AdipoR2 suggest that AdipoQ may broadly interact with the extracellular face as opposed to the carboxy-terminal tail of the receptors (Tanabe et al., 2015). The findings to date suggest that even proteins with low similarity to AdipoQs may be ligands for AdipoR in arthropods.

Although two paralogs, AdipoR1 and AdipoR2, have been identified in vertebrates, only a single AdipoR gene has been identified in all arthropods investigated to date, including insects and crustaceans. In vertebrates, AdipoR1 is ubiquitously expressed and most abundantly in skeletal muscle, whereas AdipoR2 is predominantly produced in liver (Kadowaki & Yamauchi, 2005). We found that the major production sites for Liv-AdipoR in *L. vannamei* are the skeletal muscles, hepatopancreas and hemocytes (Fig. 4). In crustaceans, the hepatopancreas is an important organ that corresponds to the liver in vertebrates. It functions as a metabolic center for digestion, absorption and storage of nutrients (Vogt et al., 1989), and the stored nutrients are transported to the skeletal muscles, gonads and other tissues during the growth and reproductive stages (Jiang et al., 2009). The observed high expression of Liv-AdipoR in hemocytes is also noteworthy (Fig. 4), as mammalian studies have also shown that AdipoR functions in regulating inflammation (Yamauchi & Kadowaki, 2013). In crustaceans, hemocytes are important cells that are involved in regulating different physiological functions, including hardening of exoskeleton, healing of cuticle damage, coagulation, carbohydrate metabolism, and protein/amino acid transportation and storage (Jiravanichpaisal, Lee & Soderhall, 2006). Collectively, the expression patterns of Liv-AdipoR are similar to those in vertebrates, and this suggests that, as single gene, it may function similar to two AdipoRs in vertebrates. Further study is warranted to determine how a single AdipoR gene can control various physiological functions in decapod crustaceans.

Since skeletal muscle in these animals exhibits high plasticity in response to a variety of physiological conditions, including molt stages or multiple limb autotomy (Covi et al., 2010; Mykles, 1997), we examined the transcriptional changes of Liv-AdipoR during the molt cycle (Fig. 4). Although we failed to identify any statistically significant difference in transcription levels of Liv-AdipoR during the molt cycle in the hepatopancreas and deep abdominal muscle (Fig. 4), it is noteworthy that several individuals had considerably high levels of Liv-AdipoR transcript (i.e., 13.3-fold higher than average value) in the D0 stage exclusively. We did not detect comparably high transcription levels in the other 30 individuals that were examined in different molting periods. This may be due to the short temporal induction of the Liv-AdipoR gene during the D0 stage. According to morphological differences of setal development in pleopods (Chan, Rankin & Keeley, 1988), the duration of the D_0_ stage ranges from 3 to 6 days, and this may be too short to detect upregulation of the gene for Liv-AdipoR based on morphological character alone. Further study should be done with much larger sample sizes to determine whether temporal upregulation of Liv-AdipoR occurs during the D_0_ stage.

After 4 weeks of our experiment, only 14.3% of the shrimp in the dsRNA-injected group had survived, which was approximately 2.8-fold higher survival than occurred in the control group. The reason for this high mortality is unclear, but the main physiological response observed was degradation of muscular protein induced by the knockdown of Liv-AdipoR. Levels of three amino acids, NH3, ornithine, and 3-methylhistidine (3-MeH), had increased significantly by 3 days after the Liv-AdipoR knockdown was induced (Table 2). NH3 and ornithine are nitrogenous wastes produced by protein catabolism. Although decapod crustaceans appear not to have a complete urea cycle, it is suggested that an alternative pathway, such as argininase activity, produces ornithine (Hartenstein, 1971; Lee & Chen, 2004). Upregulation of two genes, mitochondrial ornithine transporter and alanine racemase, may occur due to a feedback response from induced amino acid catabolism. In addition, the transcriptional induction of molecular chaperones is noteworthy, including that of E3 ubiquitin-protein ligases (ID 2976, ID 562, ID 11991, ID 5125) and heat shock protein 90 (HSP 90; ID 8317) (Table 3). Ubiquitin proteins and Hsp 90 were the most well-known chaperone proteins we identified, and these play important roles in protein turnover as a physiological response to environmental changes. In particular, E3 ligases are among the best-known regulators of skeletal muscle atrophy, and they play important roles in triggering atrophy in mammals (Bodine & Baehr, 2014). The E3 ligases observed in this study will be important markers for learning about the regulatory mechanism of muscle atrophy in crustaceans. In addition to the transcriptional changes, the increased plasma levels of 3-MeH that we observed are additional evidence that Liv-AdipoR knockdown induces muscle atrophy (Table 2). The E3 ligase 3-MeH, which is derived from the contractile proteins actin and myosin, is a well-known marker of muscle breakdown (Elia et al., 1981; Long et al., 1977; Munro & Young, 1977; Sheffield-Moore et al., 2014). Those results indicate that Liv-AdipoR is important to maintain the skeletal muscle and its deficiency causes muscle protein degradation.

Sequence-specific dsRNA injection was demonstrated to be the most successful strategy for gene-specific RNA knockdown in most decapod crustaceans, and provides an alternative means of increasing knowledge about their physiology (Sagi, Manor & Ventura, 2013). Although RNAi technique has been applied to understand various aspects of physiology, including growth and development (De Santis et al., 2011; Glazer et al., 2010; Soñanez-Organis, Racotta & Yepiz-Plascencia, 2010), immunity (Robalino et al., 2007), and reproduction (Nagaraju, Rajitha & Borst, 2011; Sathapondecha et al., 2011; Treerattrakool, Panyim & Udomkit, 2011), the cellular mechanism of this method is still not clearly understood in decapod crustaceans, and the doses, types and sizes of RNA vary for each gene examined. In the present study, transcription of Liv-AdipoR was suppressed by up to 95% in thoracic muscle and deep abdominal muscle, whereas its suppressive effects in the hepatopancreas differed according to the dsRNA concentration injected (Fig. 5). Although the mechanism of RNAi is not fully understood and has several limitations for practical application in decapod crustaceans (e.g., dose, tissue-specificity, or delivery), there is no doubt that this strategy can help to expand our knowledge about the physiological phenomena of these animals, which are considered a non-model system.

We found that most genes upregulated by the Liv-AdipoR knockdown can be classified into four major cellular functions: RNA-editing and transcriptional regulators, molecular chaperones, metabolic regulators, and channel proteins (Table 3). Given that we screened only those with greater than 10-fold change in transcriptional levels, the contigs obtained may provide only fragmented information. However, given the limited genomic information available for decapod crustaceans, characterization of the genes most obviously changed would be an effective initial strategy in non-model animals. RNA-editing and transcriptional regulators were among the most dramatically upregulated genes we observed. Among them, NEDD4-binding protein of the MutS family proteins (ID 2010) was identified as the most highly induced gene (Table 3). This protein has been identified in virtually all organisms from bacteria to humans, and plays central roles in DNA mismatch repair and recombination (Diercks et al., 2008). In addition, argonaute-2 (Ago-2, ID 662) encodes a protein that interacts with the RNase III family endonuclease known as Dicer, which mediates long double-stranded RNA into small interfering RNAs (Meister, 2013), that can be further used for positive control of RNAi in decapod crustaceans. In addition, RNA-directed DNA polymerase, NFX1-type zinc finger transcription factors, and other RNA-editing proteins should be further studied to better understand the mechanism/s involved in RNAi. In mammals, it is known that introduction of too much siRNA can result in non-specific transcriptional upregulation, as innate immune responses (Whitehead et al., 2011) and those highly-upregulated genes (MutS family proteins or Ago-2 ) can be the result of nonspecific induction as opposed to sequence-specific interference. However, comparison of the transcriptomic data induced by the other genes, such as Liv-MSTN/GDF11, reveals a totally different transcriptomic change, which suggests that the upregulated or downregulated genes could not have been the result of non-specific immune responses (Data S1).

Molecular chaperones were second most highly upregulated genes (Table 3). As explained previously, E3 ligases are among the well-known regulators of skeletal muscle atrophy (with Hsp 90 the best-known chaperone protein), and these molecules play important roles in protein turnover as a physiological response to environmental changes. Of the genes involved in metabolic regulation, we observed that two which are involved in amino acid metabolism, proline dehydrogenase 2 (ID 29409) and alanine racemase (ID 1435), were distinctly upregulated. Proline dehydrogenase 2 converts proline to delta-1-pyrroline-5-carboxylate, which is the first step of the pathway in which proline is degraded to glutamate as part of amino-acid degradation (Crabtree & Newsholme, 1970). Alanine racemase (ID 1435) is a PLP-dependent enzyme that catalyzes the interconversion of D- and L-alanine. Aquatic crustaceans and some bivalve mollusks contain a large amount of free D-alanine (i.e., up to 100 mmol/g wet weight) in their tissues (Abe et al., 2005). Although D-alanine is one of the major compatible osmolytes responsible for intracellular isosmotic regulation, induction of alanine racemase may be involved in amino acid catabolism through interference with Liv-AdipoR. In addition to genes involved in amino acid metabolism, the glycolytic enzyme triosephosphate isomerase, or TIM (ID 8958), was identified as an upregulated gene. This protein catalyzes the interconversion of dihydroxyacetone phosphate (DAP) and D-glyceraldehyde-3-phosphate (GAP), but exhibited only 65% amino acid sequence identity to the previously known TIM in *L. vannamei* (AFT92034). This requires further study. Carboxylesterase (ID 2112) is involved in lipid metabolism; however, carboxylesterases act on a variety of substrates, from water-soluble short acyl chain esters to long chain triacylglycerols, and the function of currently identified carboxylesterases should be characterized further. One interesting finding is that granulin homologs were identified as genes upregulated by the Liv-AdipoR knockdown. Granulins (ID 6271) are a family of secreted glycosylated peptides that are induced by a high-fat diet and responsible for insulin resistance, adipocyte hypertrophy, and obesity (Matsubara et al., 2012). It is interesting to know that granulin homologs were identified as genes induced by the Liv-AdipoR knockdown in a decapod crustacean. Relationships between newly identified granulin homologs and Liv-AdipoR in decapod growth should be investigated.

Of the channel and receptor proteins we observed, three proteins were involved in transporting carbohydrates (sugar transporter), proteins (mitochondrial ornithine transporter 1) and nucleosides (nucleoside transporter 3), and one was the cation channel, nucleoside transporter 3 (Table 3). Given that we identified increased levels of ornithine, NH3, and 3-MeH in association with Liv-AdipoR knockdown, these may play roles in transporting metabolites related to muscular protein degradation. Further studies are needed to elucidate the mechanism of Liv-AdipoR in maintaining skeletal muscle in decapod crustaceans.

Relatively lower numbers of downregulated genes were identified (Table 4). First, we identified that Liv-AdipoR transcripts were suppressed by more than 11-fold, which indicated that RNAi was successful. The most strongly downregulated gene was masquerade (mas)-like protein (ID 39468), which contains a trypsin-like serine protease domain at its C-terminal region. This protein was originally known as the pattern-recognition protein-activating prophenoloxidase (proPO) immune system in insects and in decapod crustaceans (Kim et al., 2002; Kwon et al., 2000). In addition, heat shock protein 21 (Hsp21), cuticle protein 6, cytochrome P450, and ribosomal L7 are known to be involved in immunity in shrimp (Huang et al., 2008; Leu et al., 2007). We also identified two transcription factors, Kruppel-homolog 1 (Kr-h1) and protein msta, as downregulated genes. Kr-h1 is a zinc finger transcription factor known to play a role in orchestrating juvenile- and ecdysone-regulated transcriptional pathways in metamorphosis and neuronal development (Minakuchi, Zhou & Riddiford, 2008; Shi et al., 2007). Protein msta (ID 18410) is a negative regulator of gene expression by methyltransferase activity. Finally, two contigs of beta-N-acetylglucosaminidase (ID 29645, 17352), which is involved in chitin degradation, were identified and the biological implications of these should be investigated.

In conclusion, this study was the first to identify and characterize the full-length cDNA encoding an AdipoR homolog from a decapod crustacean (Liv-AdipoR). Based on the genomic and biochemical experiments conducted, Liv-AdipoR appears to be involved in regulating energy expenditure in these animals. Although we did not observe any change in glucose level in response to the Liv-AdipoR knockdown, we were able to identify that Liv-AdipoR is important for maintaining skeletal muscle fiber. In addition, we made novel discoveries of some interesting genes involved in various physiological processes, including RNA-editing, metabolic regulation, transportation, and immune responses. These findings will help to expand knowledge of the physiology of decapod crustaceans.

##  Supplemental Information

10.7717/peerj.2221/supp-1 Data S1 Raw data Excel file.

## Additional Information and Declarations

### DNA Deposition

The following information was supplied regarding the deposition of DNA sequences: Liv-AdipoR, GenBank number: AKV16260.

### Data Availability

The following information was supplied regarding data availability: The raw data can be found at Figshare: 10.6084/m9.figshare.3428114.
